# Detection of sex in adults and larvae of *Leptinotarsa decemlineata* on principle of copy number variation

**DOI:** 10.1038/s41598-022-08642-x

**Published:** 2022-03-17

**Authors:** Vladimíra Sedláková, Pavel Vejl, Petr Doležal, Jakub Vašek, Daniela Čílová, Martina Melounová, Petr Sedlák

**Affiliations:** 1 Department of Genetics and Breeding, Faculty of Agrobiology, Food and Natural Resources, Czech University of Life Sciences Prague, Kamýcká 129, 16500 Prague 6, Suchdol, Czech Republic; 2 Department of Potato Protection, Potato Research Institute Havlíčkův Brod, Ltd., Dobrovského 2366, 58001 Havlíčkův Brod, Czech Republic

**Keywords:** Structural variation, PCR-based techniques, Entomology

## Abstract

The identification of sex in larvae of insects is usually challenging or even impossible, while in adults the sexual dimorphism is usually evident. Here, we used copy number analysis to develop a method of sex detection in Colorado potato beetle (*Leptinotarsa decemlineata*), which has an X0 sex determination system. The X linked gene *LdVssc* and autosomal gene *LdUBE3B* were identified as appropriate target and reference loci, respectively. The copy numbers (CNV) of *LdVssc* in males and females were estimated using standard droplet digital PCR (ddPCR) and real-time PCR (qPCR). With both methods, CNVs were bimodally distributed (BA_ddPCR_ = 0.709 and BA_qPCR_ = 0.683) with 100% ability to distinguish females from males. The use of qPCR-based sex detection in a broad collection of 448 random CPB adults showed a perfect association (Phi = 1.0, *p* < 0.05) with the true sexes of adults, with mean CNV in females of 2.032 (SD = 0.227) and 0.989 in males (SD = 0.147). In the collection of 50 random 4th instar larvae, 27 females and 23 males were identified, consistent with the expected 1:1 sex ratio (*p* = 0.689). The method is suitable for sexing in all stages of ontogenesis. The optimal cost-effective application of the method in large populations requires the DNA extraction using CTAB, the qPCR assay in one biological replicate and three technical replicates of each marker, and the use of one randomly chosen male per run to calibrate calculation of CNV.

## Introduction

Insects (*Insecta*) represent the largest group of animal species with worldwide distributions^1^. Most insects reproduce sexually and, apart from occasional developmental anomalies, they appear to be obligately gonochoristic^2,3^. The tremendous diversity of insects is also associated with great diversity of sex determining mechanisms both within and between systematic groups^4,5^. Sex determination often involves sex chromosomes (gonosomes), which have evolved independently many times^6^, and are important on other biological processes, such as genomic imprinting conflicts and speciation^7^. Gonosomes evolve from homologous autosomes that acquired a sex determining locus^6^. Sex determination is a complex developmental gene cascade, which starts on a primary sex determining gene, that controls downstream genes and differs across different insects, and ends in a terminal gene that is conserved in different insects^8,9^.

Male heterogamety (XY or X0, if sex chromosomes are present) is the commonest sex determination in insects^10^, currently documented in 77% of species investigated^4^. The Colorado potato beetle (CPB—*Leptinotarsa decemlineata* Say, 1824) studied here, is one of the most successful globally invasive insect herbivores, with high reproductive potential^11^. The male chromosome number of the species is 2n = 32 + X0 or 2n = 34 + X0^12^. The species belongs to the order *Coleoptera*, in which 771 of the 4960 species (15%) have X0 sex determining systems^10^. The occurrence of X0 males is not equally distributed in beetle families, but its frequency in *Chrysomelidae* is estimated at 16%^13^. The evolution of X0 systems is not well understood, but the "fragile Y" hypothesis^14,15^ proposes that recurrent selection to reduce recombination between the X and Y chromosome leads to genetically degenerated Y chromosomes with physically small pseudoautosomal regions (PAR); as these regions are required for regular meiotic division of gonosomes, reduction in size may increase aneuploid gamete production, leading to loss of the Y once it has degenerated to the point that it no longer carries any essential genes.

In *Coleoptera*, adults are morphologically sexually dimorphic^16,17^, but dimorphism is mostly absent earlier in ontogeny^18,19^, including the CPB. The sex of CPB adults can be reliably identified based on differences in the last abdominal sternite^16^. However, there is no morphological marker of sex for the four larval instars. Here, we show that sexing of CPB larvae is possible based on copy number variation (CNV) of X-linked genes (present in one copy in males, and two in females), relative to an autosomal reference gene^20^ (with two copies in both sexes). We tested the CPB *LdVssc* gene as a target locus, as it has been shown to be X-linked^21^. This gene is a candidate for pyrethroid resistance^22^ that encodes a voltage-sensitive sodium channel protein, and is highly conserved across *Insecta* and within populations of different insect species^23^. As a potential reference gene, an autosomal single-copy gene *LdUBE3B*, encoding ubiquitin-protein ligase E3B, was chosen. This gene was localized on linkage group 09 of the beetle species *Tribolium castaneum* Herbst, 1797^24^.

CNV can be reliably detected using droplet digital PCR (ddPCR). However, the hardware is prohibitively expensive for widespread use^25^. The main objective of our work was therefore to develop method of sex detection in CPB larvae based on CNV using standard quantitative real-time PCR (qPCR), to compare its reliability with CNV detection using ddPCR, and to identify possible factors influencing the results of such analysis.

## Material and methods

### Sample collections and sex detection of adults

Adults and larvae of CPB used in the research were collected in 16 localities of the Czech Republic in July 2019. The individuals collected were separately placed into 1.5 polypropylene tubes, frozen in liquid nitrogen, and stored in a deep freezer at − 80 °C (SANYO).

The method for molecular sex identification based on CNV was developed and optimised on a collection of adult CPB (collection A, with 10 females (F1–F10) and 10 males (M1–M10)) collected in potato fields of the Experimental station of Czech University of Life Sciences in Prague—Suchdol. An additional set of randomly collected adults from 15 other localities in the Czech Republic (collection B), with 234 females and 214 males, was used to test the reliability of the method. The true sexes of adults were identified using morphological differences in the last visible abdominal sternite, following Khidhir and Mustafa^16^. A third sample of 50 random 4th instar larvae collected in Prague (collection C) was used to test the accuracy of the new sexing method in larvae.

### DNA extraction and quantification

To check flexibility of the molecular sexing method, two different methods of DNA extraction from various tissue materials were used.

Genomic DNA from adults in collection A was isolated in two biological replicates (I and II), from caput and thorax with legs, respectively. This was performed using a NucleoSpin® Tissue Kit (Macherey–Nagel, Germany) following an animal tissue support protocol. Prior to the DNA extraction, tissues of both replicates were homogenized using Precellys Lysing Kit for hard tissue (Bertin Instruments, France) at maximum speed in Minilys (Bertin Instruments, France) for 180 s.

Single extractions of genomic DNA of samples from collections B and C used an optimised CTAB method following Chen et al.^26^. The CTAB buffer consisted of 2% (w/v) CTAB diluted in 100 mM Tris–HCl, 20 mM EDTA, and 1.4 M NaCl; 0.2% (v/v), β-mercaptoethanol was added immediately before use^27^. 200 µL of CTAB buffer and 1 µL β-mercaptoethanol were added to each tissue sample in 1.5 mL polypropylene tubes, represented by 4 legs of adult or by caput with thorax of larva. The samples were homogenized using a sterile glass rod and then 2 µL of RNase A 100 mg mL^−1^ solution (Qiagen, Germany) were added. After incubation at 37 °C for 1 h, 2 µL of Proteinase K 20 mg mL^−1^ solution (Macherey–Nagel, Germany) was added, followed by further incubation at 50 °C for 1 h. The homogenate was then extracted with 200 µL of Roti-Phenol/chloroform/isoamyl alcohol (Carl Roth GmbH, Germany), vortexed vigorously and centrifuged (14,000×*g*, 10 min). The supernatant was transferred into a new 1.5 mL tube to which 200 µL chloroform/isoamyl alcohol (24:1) was added, and the mixture was then vortexed and centrifuged (14,000×*g*, 10 min). The supernatant was transferred into a new 0.5 mL tube. To precipitate DNA, an equal volume of chilled isopropanol was added, and the tube was very gently mixed. After the formation of a precipitate, the samples were incubated at − 20 °C for 1.5 h and centrifuged (14,000×*g*, 10 min). The pellet was washed twice with 200 µL of absolute ethanol, and centrifuged (14,000×*g*, 10 min). The pellet was dried in a thermoblock at 45 °C for 30 min. The dry pellet was then dissolved in 50 µL of 1 × TE buffer overnight.

DNA samples were quantified with a NanoPhotometer (Implen, Germany) and diluted with PCR grade water (Sigma, Germany) to a concentration of 5 ng µL^−1^.

### Design of primers

The gonosomal *LdVssc* and autosomal *LdUBE3B* genes were used as the target and reference genes, respectively, because the complete sequences of both genes were identified in whole genome sequencing data of *L. decemlineata* and enabled to design primers of equivalent parameters needed for amplification at the same conditions. To prevent problems with specificity across population, the specific primer pairs were located in the exon regions of each gene using Primer3 Input 0.4.0 Program^28^ and Primer-BLAST (National Center for Biotechnology Information). Source data used to design the primers are specified in Table 1 with the final primer sequences for both genes.

**Table 1:** Markers designed for CNV-based sex identification system in CPB.

Gene	Primer	Sequence (5′–3′)	Sequence	Primer position	Amplicon size (bp)
*LdVssc*	LdVssc-F	AGAATCATGGATTGTCCGAAGGTT	Ldec_2.0 scaffold2248 (NW_019291534.1)	14,058–14,081	243
	LdVssc-R	GAGGGTGGTAAGAGTGGCAAAAGT		14,277–14,300	
*LdUBE3B*	LdUBE3B-F	AACAACTGCAGCATCTGAAACTCC	Ldec_2.0 scaffold202 (NW_019289582.1)	72,367–72,390	250
	LdUBE3B-R	TACGGCTTTGAACACTTTGACACA		72,593–72,616	

### Droplet digital PCR (ddPCR) assay

The ddPCR mix with a total volume of 25 μL, prepared for each marker separately, consisted of 12.5 μL of ddPCR EvaGreen Supermix (Bio-Rad, USA), 23 ng of genomic DNA and 40 nM each primer (Table 1). 20 μL of the ddPCR mix and 70 μL of Droplet Generation Oil for EvaGreen® (Bio-Rad, USA) were mixed using QX200™ droplet generator (Bio-Rad, USA). Samples (40 μL) containing droplets were immediately transferred into a 96-well plate (Bio-Rad, USA), sealed with aluminium foil, and placed in the deep well block of a T100™ Thermal Cycler (Bio-Rad, USA). The two-step PCR amplification consisting of the initial denaturation (95 °C, 300 s) followed by 40 cycles of denaturation (95 °C, 30 s) with annealing + extension step (60 °C, 60 s), followed by a final incubation sequence: 1 × (4 °C, 300 s), 1 × (90 °C, 300 s) and 1 × (10 °C, 300 s). The ramp rate between each step was 2 °C s^-1^. After amplification, the plate was incubated at 23 °C for 15 min and then transferred to QX200 reader (Bio-Rad, USA). Positive and negative droplets were counted using the QuantaSoft Version 1.7.4.0917 software (Bio-Rad, USA). The CNV of target *LdVssc* gene was calculated using Eq. (1).

1 $${\text{CNV}}_{LdVssc} = 2 \times { }\frac{{{\text{c}}\left( {LdVssc} \right)}}{{{\text{c}}\left( {LdUBE3B} \right)}}$$

where c is the concentration of template molecules (template copies μL^−1^).

### Real-time PCR assay

The qPCR mixes with a total volume of 10 μL, were prepared separately for each marker in technical triplicates. These consisted of 5.0 μL of FastStart Essential DNA Green Master (Roche, Switzerland), 20 ng of genomic DNA and 0.5 μM each primer. The amplification and melting temperature analysis were performed in thin wall hard-shell PCR plates (Bio-Rad, USA) using CFX Connect Real-Time System (Bio-Rad, USA) under the following conditions: initial denaturation (95 °C, 600 s), 40 cycles of denaturation (95 °C, 20 s), annealing (60 °C, 20 s) and extension (72 °C, 20 s). Ramp rate between each step was 5 °C s^-1^. During the subsequent melting analysis, the temperature changed from 65.0 to 95.0 °C by increments equal to 0.5 °C s^-1^. The efficiency (E) of both primer pairs was determined using dilution series with 20.0, 10.0, 5.0, 2.5, and 1.25 ng of genomic DNA per 10 μL reaction. The values of the variables E [%], R^2^, slope, and y-intercept (y-int) were determined using the Bio-Rad CFX Maestro software (Bio-Rad, USA). The difference between the slope values of curves obtained by plotting log inputs versus ΔCt (Ct_*LdVssc*_ − Ct_*LdUBE3B*_) was assessed according to Larionov et al.^29^.

The optimization and validation of the qPCR assay using the CPB collection A adults was designed in two biological replicates and three technical replicates. One adult male M1 was used as a calibrator. The verification of the method on DNA samples of collections B and C extracted by CTAB used one biological replicate and three technical replicates, when one random male (a putative male larva) was used as the calibrator for each PCR run.

The copy number value based on the REST© algorithm according to Pfaffl et al.^30^ was calculated using a male as a calibrator (Eq. 2).

2 $${\text{CNV}}_{LdVssc} = \frac{{{\text{E }}\left( {LdVssc} \right)^{{\Delta {\text{Ct }}\left( {LdVssc} \right)}} }}{{{\text{E }}\left( {LdUBE3B} \right)^{{\Delta {\text{Ct }}\left( {LdUBE3B} \right)}} }}$$

where E: Efficiency of primers, *∆*Ct: Difference in cycle threshold (Ct) between the mean of technical replicates of the calibrator and the mean of technical replicates of the tested sample.

### Sequencing of LdVssc and LdUBE3B amplicons

DNA fragments of one male and one female from collection A, were amplified by qPCR and sequenced in three replicates. Amplicons were extracted from 1.5% agarose gel using a MiniElute PCR Purification Kit (Qiagen, Germany), amplified using BigDye Terminator v 3.1 Kit (Life Technologies, USA) and analysed with a ABI 3730xl DNA Analyser (Life Technologies, USA). The sequences obtained were compared with published data using the program BioEdit version 7.0.5.3^31^ and deposited in the NCBI international nucleotide database.

### Statistical evaluation of experiments

The distributions of CNV values obtained for all datasets were tested for bimodality^32^ using the statistical program R v4.1.0^33^. In the optimization process, the equality of the CNV values of biological replicates detected by ddPCR and qPCR was evaluated by paired sample *t*-tests, after confirming the normality of differences by the Shapiro–Wilk test. The results of molecular sex identification of all adults were compared with the true sex using association coefficient $\left( \Phi \right)$. The sex ratio detected in the collection of larvae by qPCR was compared with the expected 1:1 ratio using a χ^2^-test. These statistical evaluations were done using the Dell Statistica Software (Dell, USA).

### Ethical approval

All of the experimental procedures were conducted in accordance with Czech legislation (Section 29 of Act No. 246/1992 Coll. on the protection of animals against cruelty, as amended by Act No. 77/2004 Coll.). We hereby declare that animal handling conducted in our study complies with the relevant European and international guidelines on animal welfare, namely Directive 2010/63/EU on the protection of animals used for scientific purposes and the guidelines and recommendations of the Federation of Laboratory Animal Science Associations. All experimental protocols were approved by the Czech University of Life Sciences Prague, Faculty of Agrobiology, Food and Natural Resources of the Czech Republic and Institutional and National Committees.

## Results

### Specificity of PCR markers of LdVssc and UBE3B genes

**Figure 1:**
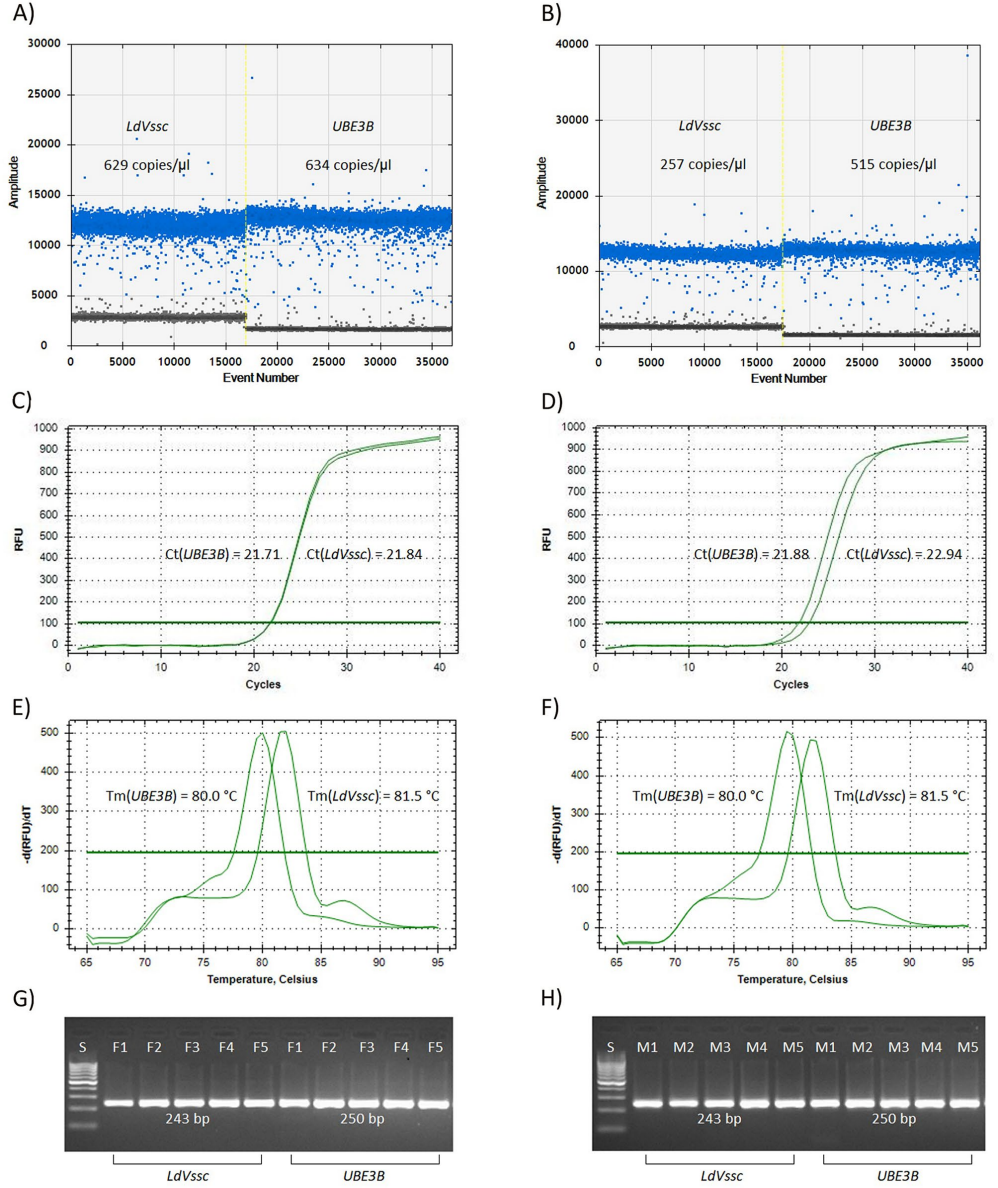
Outputs of ddPCR and qPCR of CNV based sex detection using markers *LdVssc* and *LdUBE3B* typically detected in female (left side) and male (right side). Counts of positive and negative droplets (**A**, **B**), qPCR Ct values (**C**, **D**), qPCR melting analysis (**E**, **F**), and electrophoretograms of *LdVssc* and *LdUBE3B* amplicons (**G**, **H**).

The electrophoretograms (Fig. 1)G,H) show that both primer pairs specifically amplified only DNA fragments of 243 bp and 250 bp, the expected sizes for *LdVssc* and *LdUBE3B* genes, respectively. The sequences of the *LdVssc* and *LdUBE3B* amplicons in the female F1 and male M1 from the collection A were deposited in the NCBI international nucleotide database under accession numbers MZ962721, MZ962722, MZ962723 and MZ962724 respectively. The sequences were identical in males and females and BLAST analysis of the *LdVssc* amplicons showed full sequence homology with the sequence NW_019291534.1, which was used to design the primers, and with various other transcripts of the gene (XR_002722894.1, XM_023167295.1, XM_023167296.1, XM_023167297.1, XM_023167298.1, XM_023167299.1, XM_023167300.1 and XM_023167301.1). Similarly, the *LdUBE3B* amplicon was fully homologous with the NW_019289582.1 sequence used to design the primers. The amplicon of the *LdUBE3B* gene contained partial sequences of exons 6 and 7 with the entire intron 6 (58 bp), and its exon region sequence was also identical with the published *LdUBE3B* mRNA sequence (XM_023174728.1). The specificity of our primers was also confirmed by melting analysis (Fig. 1E,F). The graphs show the melting curves typical for both genes in both male and female samples. The melting temperatures (T_m_) 81.5 °C and 80.0 °C were determined for both *LdVssc* and *LdUBE3B* amplicons respectively.

### Optimization of ddPCR and qPCR assays

Table 2 shows the parameters characterizing ddPCR in collection A, including descriptive statistics, and the CNV values are in Table 3. The analysis outputs (Fig. 1A,B) from QuantaSoft Version 1.7.4.0917 (Bio-Rad, USA) demonstrated that we had achieved an optimal setup of the assay, since most counted droplets were classified as positive or negative, and few droplets were detected in the “rain region”.

**Table 2:** Summary of results of ddPCR and qPCR in the collection A.

	Target (*LdVssc* gene)								Reference (*LdUBE3B* gene)							
	Females				Males				Females				Males			
	Min	Max	$\overline{{\text x}}$	SD	Min	Max	$\overline{{\text x}}$	SD	Min	Max	$\overline{{\text x}}$	SD	Min	Max	$\overline{{\text x}}$	SD
**ddPCR assay**																
Number of positive droplets	6999	7058	7011	281	3405	3491	3457	138	6981	7051	7021	279	6821	6990	6914	281
Number of negative droplets	10,653	10,801	10,787	438	14,103	14,295	14,193	554	10,636	10,795	10,704	431	10,715	10,873	10,784	427
Number of template copies per 1 μL	623	635	627	26	249	263	259	12	621	632	624	24	499	521	509	19
**qRT-PCR assay**																
Ct	20.93	22.12	21.43	1.11	22.86	24.12	23.51	1.19	20.81	22.08	21.51	1.13	20.95	22.14	21.40	1.10

**Table 3:** Comparison of CNV values detected by ddPCR and qPCR in the collection A.

Female	ddPCR assay			qPCR assay			Male	ddPCR assay			qPCR assay		
	Replicate		$\overline{{\text x}}$	Replicate		$\overline{{\text x}}$		Replicate		$\overline{{\text x}}$	Replicate		$\overline{{\text x}}$
	I	II		I	II			I	II		I	II	
F1	2.011	2.009	2.010	2.024	1.985	2.005	M1	0.968	0.979	0.974	1.012	1.026	1.019
F2	1.993	1.987	1.990	1.899	1.966	1.933	M2	1.021	1.011	1.016	0.931	0.975	0.953
F3	2.007	2.011	2.009	2.057	2.096	2.077	M3	0.981	0.987	0.984	1.017	1.002	1.010
F4	1.975	1.978	1.977	2.041	1.987	2.014	M4	1.011	1.024	1.018	1.025	0.999	1.012
F5	2.018	2.009	2.014	1.874	1.993	1.934	M5	1.001	0.998	1.000	0.899	0.891	0.895
F6	1.971	1.965	1.968	1.941	2.051	1.996	M6	1.014	1.012	1.013	0.916	1.012	0.964
F7	1.983	1.991	1.987	2.065	1.997	2.031	M7	0.999	0.991	0.995	1.036	1.036	1.036
F8	2.019	2.016	2.018	1.873	2.014	1.944	M8	1.021	1.009	1.015	0.877	1.023	0.950
F9	1.971	1.968	1.970	1.885	1.915	1.900	M9	0.987	0.995	0.991	0.955	0.881	0.918
F10	1.993	1.971	1.982	1.912	1.888	1.900	M10	1.021	1.036	1.029	0.931	0.989	0.960
$\overline{{\text x}}$			1.992			1.973	$\overline{{\text x}}$			1.003			0.972
SD			0.019			0.071	SD			0.018			0.056

Comparable parameters were found for our optimized qPCR protocols, slope = − 3.324 (− 3.341), y-int = 38.476 (36.528), R^2^ = 0.985 (0.998) and E = 99.9% (99.2%), for both the target *LdVssc* and reference *LdUBE3B* genes. Since the difference between the slope values (0.017) was between − 0.1 to + 0.1, the CNV values could be determined by processing of raw Ct data, as suggested by Larionov et al.^29^. The qPCR-based Ct and CNV values detected specifically for collection A are presented in Tables 2 and 3, respectively. The optimization of method resulted in characteristic sets of qPCR curves for both studied genes (Fig. 1C,D). In the case of females (Fig. 1C), the curves have almost identical positions, resulting in ΔCt value approximately equal to 0.0, indicating the same copy numbers of target and reference gene. The shifted curves in males (Fig. 1D) correspond with a ΔCt value of approximately 1, indicating the expected halved quantity of target template in the sample, compared with the reference gene.

### Statistical evaluation of the experiments

**Figure 2:**
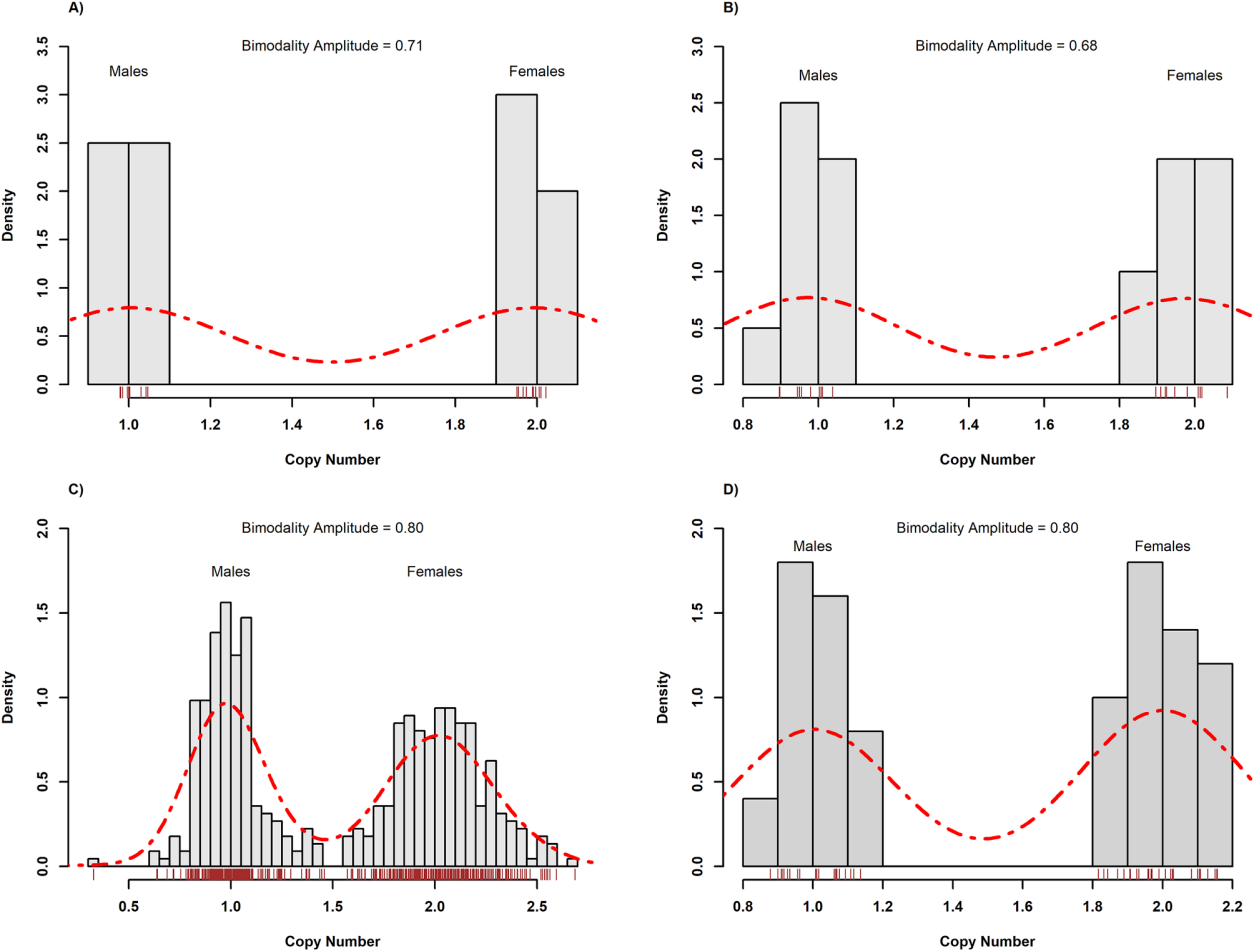
Bimodal distribution of copy number values (CNV) documents the ability of ddPCR and qPCR to distinguish females and males in: the collection A using ddPCR (**A**) and qPCR (**B**), the collection B using qPCR (**C**), and the collection C by qPCR (**D**).

In collection A, the CNV values had a bimodal distribution, as characterized by the bimodality amplitude (BA) for both the ddPCR (BA = 0.709) and the qPCR (BA = 0.683) assays, as documented by histograms (Fig. 2A,B). The bimodality corresponded with the true sex of adults, with average CNV values of females and males of approximately 2 and 1, respectively (Table 4). The CNV values associated perfectly with individuals’ true sexes ($\Phi$ = 1.0, *p* < 0.05). Paired sample t-tests detected no significant differences in the mean CNV values of biological replicates using either ddPCR (*t* = 0.430, *p* = 0.672) or qPCR (*t* = − 1.809, *p* = 0.086). Normality of differences between CNV values of the biological replicates (required for the paired *t*-test) was confirmed by the Shapiro–Wilk test (W = 0.972, *p* = 0.802; W = 0.953, *p* = 0.414) for the ddPCR and qPCR methods, respectively. These results confirmed that both our sex prediction approaches are not affected by the biological material used to extract the genomic DNA.

**Table 4:** Bimodality Amplitude (BA) values and other descriptive statistics of CNV values in all collections.

Assay	BA value	Sex (true)	Sex (CNV based)	N	Mean	SD	CI mean 95%	Min	Max	CV
**Collection A**										
*ddPCR*	0.709	F	F	10	1.990	0.019	0.013	1.970	2.020	0.009
		M	M	10	1.000	0.017	0.012	0.974	1.030	0.017
qPCR	0.683	F	F	10	1.973	0.060	0.043	1.900	2.077	0.030
		M	M	10	0.972	0.046	0.033	0.895	1.036	0.048
**Collection B**										
qPCR	0.795	F	F	234	2.032	0.227	0.029	1.444	2.673	0.112
		M	M	214	0.989	0.147	0.020	0.320	1.418	0.149
**Collection C**										
qPCR	0.799	–	F	27	2.000	0.101	0.040	1.827	2.174	0.050
		–	M	23	1.004	0.084	0.036	0.876	1.124	0.084

In collections B and C, the results agreed with those detected for collection A. The CNV values detected by qPCR were comparable with values obtained in collection A and again showed bimodal distributions in males (Table 4 and Fig. 2C,D). True sex associated perfectly with the CNV values in collection B ($\Phi$ = 1.0, *p* < 0.05). In collection C, 27 females and 23 males were predicted by qPCR, in agreement with the expected 1:1 sex ratio (χ^2^ = 0.16, *p* = 0.689).

## Discussion

The study confirmed that CNV based sexing is possible using the X-linked *LdVssc* gene as a target and *LdUBE3B* gene as a reference. The target gene was detected in single copy in all 224 males (X0) and all 244 females (XX) were assessed as having two copies, after normalisation with the reference gene. The *LdUBE3B* gene required validation as a reference gene. Its genomic location in CPB was unknown (it is in an assembled unplaced genomic scaffold of the species, Ldec_2.0 scaffold202 (NW_019289582.1)), but its presence on autosomal linkage group 9 of *Tribolium castaneum* (NCBI gene ID: 659339) suggested that it could potentially be a suitable reference gene. Our results showed that the efficiencies of our primer pairs for both this gene and the target gene were comparable. Our results also documented that the *LdUBE3B* gene does not show sign of copy number variation (whereas the approximate mean copy number values for the *LdVssc* target gene in all experimental collections behaved as expected, with a bimodal distribution (Fig. 2) with peaks at CNV = 1 and CNV = 2, in males and females respectively, as just described). The *LdUBE3B* gene was therefore confirmed as an autosomal single-copy gene in CPB.

One of the expected options of the optimization was to design a valuable, flexible, repeatable, and cost-effective method. The DNA extraction kit used in the optimization stage is not cost-effective for large numbers of population samples. Our analyses of collections B and C confirmed the flexibility of the method, because the results of sexing in DNA samples extracted using the cheaper CTAB method were consistent with those in collection A. The results show that both the amount of tissue and the quality of the DNA samples were sufficient for valuable analyses of extensive population samples. Both CNV detection systems, ddPCR and qPCR, gave statistically comparable results, although the ddPCR yielded more accurate CNV values. However, in agreement with Martins et al.^25^, qPCR can be recommended as an affordable method for extensive sample sets, although three technical replicates for each marker and sample are recommended, in agreement with Svec et al.^34^.

The greater variability of CNVs in collection B using qPCR (Fig. 2C and Table 4) may be related to the number of independent DNA samples, with individual quality differences. The set of 448 samples of adults required 28 independent runs, and an effect of different qPCR runs was also detected^34,35^. Although the CN values in each run were correctly calculated using a male individual as an internal calibrator, comparisons between runs, and calculation of correct CNVs using a single universal calibrator, was almost impossible. Technically, the use of a single individual from a population as a universal calibrator for each independent run is also not realistic for large sets or very small biological samples such as 1st instar larvae, which can yield insufficient DNA amounts for tens of replicates. For these reasons, a randomly chosen male from run is recommended used as a calibrator, and the CNVs should be calculated and interpreted individually for each run. Especially for prediction of sex in larvae, the individuals with lowest ΔCt were more probably males than females. This is likely an optimal solution for majority of insect species with regular occurrence of males, because an absence of males is unlikely in colonies of larvae in such case.

Despite these difficulties, qPCR sex determination associated perfectly with the true sexes of adults, giving very good prospect for predicting the sexes of each instar CPB larvae. Our tests in a set of 50 larvae confirmed that the method gave comparable results and works on the same principle as in adults. There is no way to identify morphologically the sex in larval stages for many insect species, an in those where this is possible, it is only for the final larval instar or pupae, to a limited extent^18,19,36–38^. The method presented here therefore opens up new possibilities in the study of sex-related topics in all ontogenetic stages across insects.

The target gene *LdVssc*, known as a candidate gene of knock-down resistance (kdr) to pyrethroids^39,40^, was also confirmed by our study as a single copy gene in relatively large samples of CPB individuals collected across all regions of the Czech Republic that are important for production of potato. Copy number values higher than 2 were never detected in our experiments, suggesting that kdr susceptibility/resistance to pyrethroids in CPB is caused only by previously presented mutations within the gene^41^ and not by increased CNV of the gene, as in corresponding gene in mosquitoes^25,42^.

In conclusion, the methodology presented was independent on the developmental stage or tissue used, and the DNA extraction method, and can be optimised for all insect species with X0/XX sex determination systems to sex samples of all ontogenetic stages. Note that, as the larvae used are destroyed, the present approach cannot be used if the sexing is needed for studies of larval characteristics. However, it should be possible to develop a non-destructive modification of the method, for applications where keeping the larvae alive is important.

## Data Availability

All obtained sequences were stored in the NCBI international nucleotide database under referred accession numbers. Unpresented datasets generated and analyzed during the current study are available from the corresponding author on reasonable request.
